# Circulating Tumor DNA and Minimal Residual Disease (MRD) in Solid Tumors: Current Horizons and Future Perspectives

**DOI:** 10.3389/fonc.2021.763790

**Published:** 2021-11-18

**Authors:** Yan Peng, Wuxuan Mei, Kaidong Ma, Changchun Zeng

**Affiliations:** ^1^ Department of Obstetrics, Longhua District Central Hospital, Shenzhen, China; ^2^ Clinical Medical College, Hubei University of Science and Technology, Xianning, China; ^3^ Department of Medical Laboratory, Shenzhen Longhua District Central Hospital, Guangdong Medical University, Shenzhen, China

**Keywords:** circulating tumor DNA, minimum residual disease (MRD), biomarker, liquid biopsy, cancer

## Abstract

Circulating tumor DNA (ctDNA) is cell-free DNA (cfDNA) fragment in the bloodstream that originates from malignant tumors or circulating tumor cells. Recently, ctDNA has emerged as a promising non-invasive biomarker in clinical oncology. Analysis of ctDNA opens up new avenues for individualized cancer diagnosis and therapy in various types of tumors. Evidence suggests that minimum residual disease (MRD) is closely associated with disease recurrence, thus identifying specific genetic and molecular alterations as novel MRD detection targets using ctDNA has been a research focus. MRD is considered a promising prognostic marker to identify individuals at increased risk of recurrence and who may benefit from treatment. This review summarizes the current knowledge of ctDNA and MRD in solid tumors, focusing on the potential clinical applications and challenges. We describe the current state of ctDNA detection methods and the milestones of ctDNA development and discuss how ctDNA analysis may be an alternative for tissue biopsy. Additionally, we evaluate the clinical utility of ctDNA analysis in solid tumors, such as recurrence risk assessment, monitoring response, and resistance mechanism analysis. MRD detection aids in assessing treatment response, patient prognosis, and risk of recurrence. Moreover, this review highlights current advancements in utilizing ctDNA to monitor the MRD of solid tumors such as lung cancer, breast cancer, and colon cancer. Overall, the clinical application of ctDNA-based MRD detection can assist clinical decision-making and improve patient outcomes in malignant tumors.

## Introduction

Liquid biopsy, defined as the analysis of cancer biomarkers in tumor-derived material extracted from cancer patients’ bloodstream, urine, pleural effusion, cerebrospinal fluid, saliva, or bile, has recently gained growing attention in cancer diagnosis and treatment owing to its many benefits and application potential. Unlike traditional tissue biopsy, liquid biopsies are non-invasive, easily repeatable, and may offer a handy insight into tumor burden and treatment response. Furthermore, the liquid biopsy may give a molecular snapshot of the primary tumor, minimizing bias in biopsy findings caused by sampling bias and intratumor heterogeneity. Nucleic acids, proteins, extracellular vesicles, and other biological components secreted into bodily fluids by cancer cells are among the analytes of liquid biopsies. Circulating tumor DNA (ctDNA), circulating tumor cells (CTCs), circulating tumor RNA (ctRNA), exosomes, proteins, and metabolites as the analytes of liquid biopsies can be identified using biomarkers such as somatic point mutations, deletions, amplifications, gene fusions, DNA methylation markers, miRNAs, proteins, or metabolites. ctDNA is a potential biomarker since it contains tumor-specific genetic and epigenetic abnormalities and may be utilized in cancer diagnosis and prognosis prediction. The fact that symptoms of many cancer types are frequently absent at an early stage has resulted in extensive research efforts to create non-invasive, reliable, and cost-effective early detection techniques for these diseases. The bulk of the presently known research on the utilization of ctDNA is concerned with mutation detection. The study of ctDNA is addressed in the context of noninvasively detecting mutations that result in resistance mechanisms and monitoring treatment and disease response in cancer patients. Because the ctDNA percentages in total cell-free DNA (cfDNA) biofluid samples are extremely low, and their levels vary depending on the type and stage of cancer, highly sensitive assays are required to identify these tiny ctDNA fractions. Over the last several years, significant progress has been made in the development of ctDNA detection methods. PCR-based sequencing, which includes real-time quantitative PCR (qPCR), and digital PCR (dPCR) methods, is an alternative method for single-locus/multiplexed tests and targeted panels, while Next Generation Sequencing (NGS)-based sequencing, which includes Tagged-Amplicon deep sequencing (TAM-Seq), CAncer Personalized Profiling by deep sequencing (CAPP-Seq), and Duplex sequencing can be applied to panels of any size (1, 2). Notably, the revolution in ctDNA-based liquid biopsies has opened up new opportunities for cancer diagnosis, prognosis, monitoring, and treatment guidance (3).

Recent improvements in sequencing technology and ctDNA analysis have enabled non-invasive monitoring of the patient disease burden and assessment of molecular targets. In many tumor types, such as lung cancer, breast cancer, colon cancer, pancreatic cancer, and bladder cancer, ctDNA has been proven to be effective in detecting MRD (4). Patients with cancer may benefit from ctDNA testing to ascertain the presence of MRD and to forecast recurrence in the postoperative setting. For individuals undergoing adjuvant chemotherapy, the non-invasive and dynamic nature of the biomarker may potentially serve as a real-time indicator of adjuvant chemotherapy effectiveness. MRD tests may be utilized not only for early relapse detection and adjuvant therapy but also for initiating and monitoring systemic treatment, as well as drug resistance genotyping (5). Overall, MRD aids in the management of cancer at all stages, including screening, guiding adjuvant treatment, predicting relapse early, initiating systemic treatment and monitoring response, and genotyping resistance.

## Clinical Utility of Circulating Tumor DNA (ctDNA)

### ctDNA Detection Methods

The amount of detectable ctDNA is determined by the tumor type, tumor load, and other biological processes such as plasma nuclease activity. cfDNA is fragmented DNA, with the overall quantity of ctDNA making up as low as 0.01% of the entire cfDNA. ctDNA-based NGS technology can identify not only somatic mutations but also copy number variation (CNVs) and structural rearrangement (6). Understanding the development of ctDNA detection technology is crucial to evaluating the clinical significance of ctDNA. When it comes to sensitivity and expense, there is always a compromise. Several techniques have been suggested to decrease the cost, errors, and background noise. Droplet digital polymerase chain reaction (ddPCR), beads, emulsion, amplification and magnetics (BEAMing), tagged-amplicon deep sequencing (TAm-Seq), cancer personalized profiling by deep sequencing (CAPP-Seq), whole-genome sequencing (WGS), and whole-exome sequencing (WES) are some of the most used ctDNA detection techniques (**Table 1**) (22, 23).

**Table 1:** Circulating tumor DNA (ctDNA) detection methods.

Technique	Method	Advantages	Limitations	Reference
Allele-specific PCR	ARMS	Easy to set-up; Lowest cost	Low sensitivity; Detect specific genomic locations	(7)
Digital PCR	ddPCR	High sensitivity; Absolute quantification	Detect specific genomic locations; Limited in multiplexing	(8, 9)
	BEAMing	High sensitivity; Relatively inexpensive	Detect only known mutations	(10, 11)
Multiplex PCR-based NGS	TAm-Seq	High sensitivity; Lower cost than other NGS methods	Detect only known mutations; Less comprehensive than other NGS method	(12, 13)
	Safe-SeqS	High sensitivity; Lower cost than other NGS methods	Less comprehensive than other NGS method	(14, 15)
Hybrid capture-based NGS	CAPP-Seq	High sensitivity; Detects multiple mutation types; Broadly applicable without personalization; Lower cost than WGS/WES; Higher sequencing depth than WGS/WES	High cfDNA input; Detect only known mutations; Less comprehensive than WGS/WES	(16, 17)
	TEC-Seq	High sensitivity; Detects multiple mutation types; Broadly applicable without personalization; Lower cost than WGS/WES; Higher sequencing depth than WGS/WES	Less comprehensive than WGS/WES	(18)
Retrotransposon-based amplicon NGS	FAST-SeqS	Rapid aneuploidy assessment with lower cost than WGS/WES	Low sensitivity and specificity; Limited to aneuploidy detection	(19, 20)
Whole-genome sequencing (WGS)	WGS	The entire genome is interrogated; Broadly applicable without personalization	Limited sequencing depth; Low sensitivity; Expensive; Limited to SCNA	(21)
Whole-exome sequencing (WES)	WES	The entire exome is interrogated; Broadly applicable without personalization	Limited sequencing depth; Low sensitivity; Expensive	(21)

PCR, polymerase chain reaction; ddPCR, droplet digital polymerase chain reaction; BEAMing, bead, emulsion, amplification, and magnetics; TAm-Seq, tagged−amplicon deep sequencing; Safe-SeqS, safe-sequencing system; CAPP-Seq, cancer personalized profiling by deep sequencing; TEC-Seq, targeted error correction sequencing; FAST-SeqS, fast aneuploidy screening test-sequencing system; WGS, whole-genome sequencing; WES, whole-exome sequencing; SCNA, somatic copy number alteration; NGS, next-generation sequencing.

The ddPCR method distributes DNA samples into hundreds to millions of water-oil emulsion droplets. The advantages of ddPCR include its excellent sensitivity for identifying mutations and its low cost for absolute quantification. In comparison to NGS-based techniques, PCR-based methods have a much shorter turnaround time, with the majority of data being returned within 72 hours, as opposed to 1 to 2 weeks for massively parallel sequencing. The ddPCR method has the disadvantage of detecting only known variants and analyzing only a limited number of variants. ddPCR offers higher sensitivity than conventional quantitative PCR or NGS and a more straightforward workflow than alternative digital PCR methods like BEAMing. According to a meta-analysis, ddPCR has a high specificity (72.1%) and acceptable sensitivity (95.6%) for detecting EGFR mutations in cfDNA, which justifies its use in clinical practice as a supplement or conditional substitute for tissue biopsy for genotyping. It also appears to have a higher sensitivity than ARMS-PCR, especially in the early stages of lung cancer (24). Furthermore, KRAS G12/G13 mutations may be detected in a tiny quantity of unamplified cfDNA utilizing a droplet digital PCR multiplex technique, which has excellent agreement with conventional mutation testing for archival tumor tissue (25). Although ARMS, ddPCR, and BEAMing have excellent sensitivity and detection capabilities for various stages of cancer, their clinical applicability is restricted since these methods can only identify known mutations (23, 26, 27).

NGS is a high-throughput technique that can search for previously unidentified variations. As more therapeutically relevant molecular targets become available, NGS becomes more important in cancer. Although whole exome or whole genome sequencing may provide more detailed genomic information, ctDNA NGS techniques in clinical usage utilize hybrid capture panels or amplicon-based NGS to provide clinically relevant information with lower cost and higher sequencing depth. In the last decade, NGS has established itself as a reliable method for sequencing DNA and collecting genetic data. NGS works by analyzing millions of short DNA sequences in parallel, then aligning them to a reference genome or assembling them from a *de novo* sequence. Tagged-Amplicon deep sequencing (TAm-seq) and CAncer Personalized Profiling by deep sequencing (CAPP-Seq) are some of the techniques that are used to apply NGS to a target panel (23, 27). The enhanced TAm-Seq technique identified mutant alleles down to 0.02% allele fraction with 99.9997% per-base specificity. Samples with the optimum quantity of DNA had 94% mutations at 0.25% -0.33% allele fraction, compared to 90% mutations in samples with lower levels of input DNA (12). The integrated digital error suppression (iDES)-enhanced CAPP-Seq technique allowed biopsy-free profiling of EGFR kinase domain mutations with a sensitivity of 92% and a specificity of 96% (28). Overall, analytical sensitivity is limited by low levels of cfDNA in the blood and sequencing artifacts. More clinical investigation of novel approaches is required to overcome these constraints. Additionally, plasma cell-free DNA methylomes could allow for non-invasive, highly sensitive, low-cost, and accurate early tumor detection and classification. cfMeDIP-seq (cell-free DNA immunoprecipitation and high-throughput sequencing) for genome-wide bisulfite free plasma DNA methylation analysis is cost-effective based on its ability to enrich CPG-rich fragments that may provide additional information (29).

### Timeline of ctDNA Development

**Figure 1:**
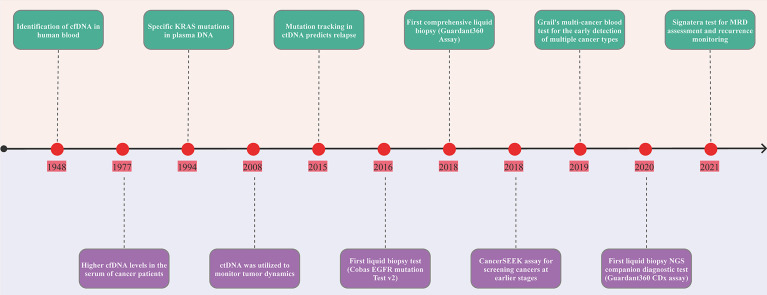
Timeline of the landmark in ctDNA analysis. The figure exhibits a timeline of selected significant milestones in ctDNA as applied to solid tumors. FDA, The United States Food and Drug Administration; MRD, minimal residual disease; ctDNA, circulating tumor DNA.

In 1948, cfDNA was identified in human blood plasma (**Figure 1**) (30). Leon et al. observed higher cfDNA levels in the serum of cancer patients in 1977 (31). Subsequent research revealed specific KRAS mutations in plasma DNA from pancreatic cancer patients in 1994 (32). Besides, circulating mutant DNA was utilized to monitor tumor dynamics in cancer patients undergoing surgery or chemotherapy in 2008 (33). A direct comparison of circulating tumor DNA with other circulating biomarkers (CA 15-3 and circulating tumor cells) and medical imaging revealed that ctDNA is an informative, specific, and highly sensitive metastatic breast cancer biomarker in 2013 (34). In 2015, detecting mutations in ctDNA was used to monitor MRD and predict the likelihood of early breast cancer recurrence, and customize adjuvant treatment strategies (35). In 2016, the United States Food and Drug Administration (FDA) approved the first “liquid biopsy test” (Cobas EGFR mutation Test V2) for patients with non-small cell lung cancer (NSCLC). In 2008, the FDA approved the first comprehensive liquid biopsy (Guardant 360 Assay) as an expedited access pathway device and the Cancer SEEK assay for cancer screening at an earlier stage as a breakthrough device. In 2019, FDA granted breakthrough device designation to Grail’s multi-cancer blood test for the early detection of multiple cancer types. In 2020, FDA approved first liquid biopsy next-generation sequencing (NGS) companion diagnostic test (Guardant 360 CDx Assay) to detect specific types of the epidermal growth factor receptor (EGFR) gene mutations in patients with NSCLC. In 2021, FDA granted two breakthrough device designations to the Signatera test for molecular residual disease (MRD) assessment and recurrence monitoring. Additionally, accumulating evidence demonstrates the usefulness of ctDNA in cancer diagnosis, prognosis, disease progression, and treatment response (36–38).

### ctDNA Analysis May Be an Alternative for Tissue Biopsy

Diagnostic tools for monitoring the molecular evolution through noninvasive techniques such as liquid biopsy are becoming accessible and will be a valuable tool for further improving personalized treatment in cancer. Especially, detection of molecular alterations utilizing ctDNA may be a viable approach for patients who do not have access to a tissue specimen or a high-quality biopsy (39). In a single-center analysis of 323 non–small cell lung cancer patients, 229 had concurrent plasma and tissue NGS or were unable to complete tissue testing. Tissue sequencing identified targetable mutations in 47 individuals (20.5%), whereas plasma sequencing identified 82 (35.8%). Moreover, 85.7% of patients treated with plasma next-generation sequencing–indicated treatment obtained a complete or partial response or stable disease (40). In patients with advanced non-small-cell lung cancer, analysis of ctDNA in blood samples may be used as a surrogate form of tumor biopsy for detecting EGFR and KRAS mutation status (41).

The ctDNA test is adequate to detect all driver DNA changes present in matched metastatic tissue in metastatic castration-resistant prostate cancer (mCRPC) patients, indicating that DNA biomarkers to guide mCRPC patient treatment based on ctDNA alone are feasible. Significant actionable alterations such as PTEN or BRCA2 loss are found in matched metastatic CRPC tissue samples, will not be missed by a well-designed ctDNA test. The excellent agreement between ctDNA and metastatic tissue biopsies in mCRPC indicates that ctDNA tests may be utilized to prognostically and predictively stratify patients (42). DNA damage repair (DDR) gene alterations identified in prostate cancer metastatic tissue or ctDNA were concordant with primary prostate cancer when clonal hematopoiesis was ruled out in genetic association analysis. Concordance in DDR gene alterations across prostate cancer samples was up to 84% (43).

The plasmaMATCH trial exhibited a significant degree of concordance across ctDNA assays and high sensitivity for mutations detected in tissue sequencing, particularly in contemporaneous advanced breast cancer samples. Advanced breast cancer patients with uncommon, potentially targetable HER2 and AKT1 mutations in ctDNA showed clinically significant responses to the HER2 inhibitor neratinib and the AKT inhibitor capivasertib, respectively, consistent with prior tissue sequencing-directed studies. These results validate the use of ctDNA testing to screen advanced breast cancer patients for rare mutations and show its clinical value (44). In a recent prospective investigation of metastatic triple-negative breast cancer, blood was shown to be a quicker and less invasive approach for molecular evaluation than tissue (45). In metastatic triple-negative breast cancer (TNBC), ctDNA was used to characterize somatic copy number alterations (SCNAs). SCNA identification is an attractive alternative to somatic mutation targeting since most tumors have SCNAs that may be easily detected using low-coverage WGS (46, 47). By comparing the SCRUM-Japan GI-SCREEN and GOZILA trials, ctDNA genotyping reduced screening time (11 vs. 33 days, P < 0.0001) and increased trial enrollment rate (9.5 vs. 4.1%, P < 0.0001) without compromising trial outcomes compared to tissue genotyping in advanced gastrointestinal cancer (48). Overall, ctDNA analysis is gaining popularity as a novel method of tumor genotyping.

### Recurrence Risk Assessment Using ctDNA

**Figure 2:**
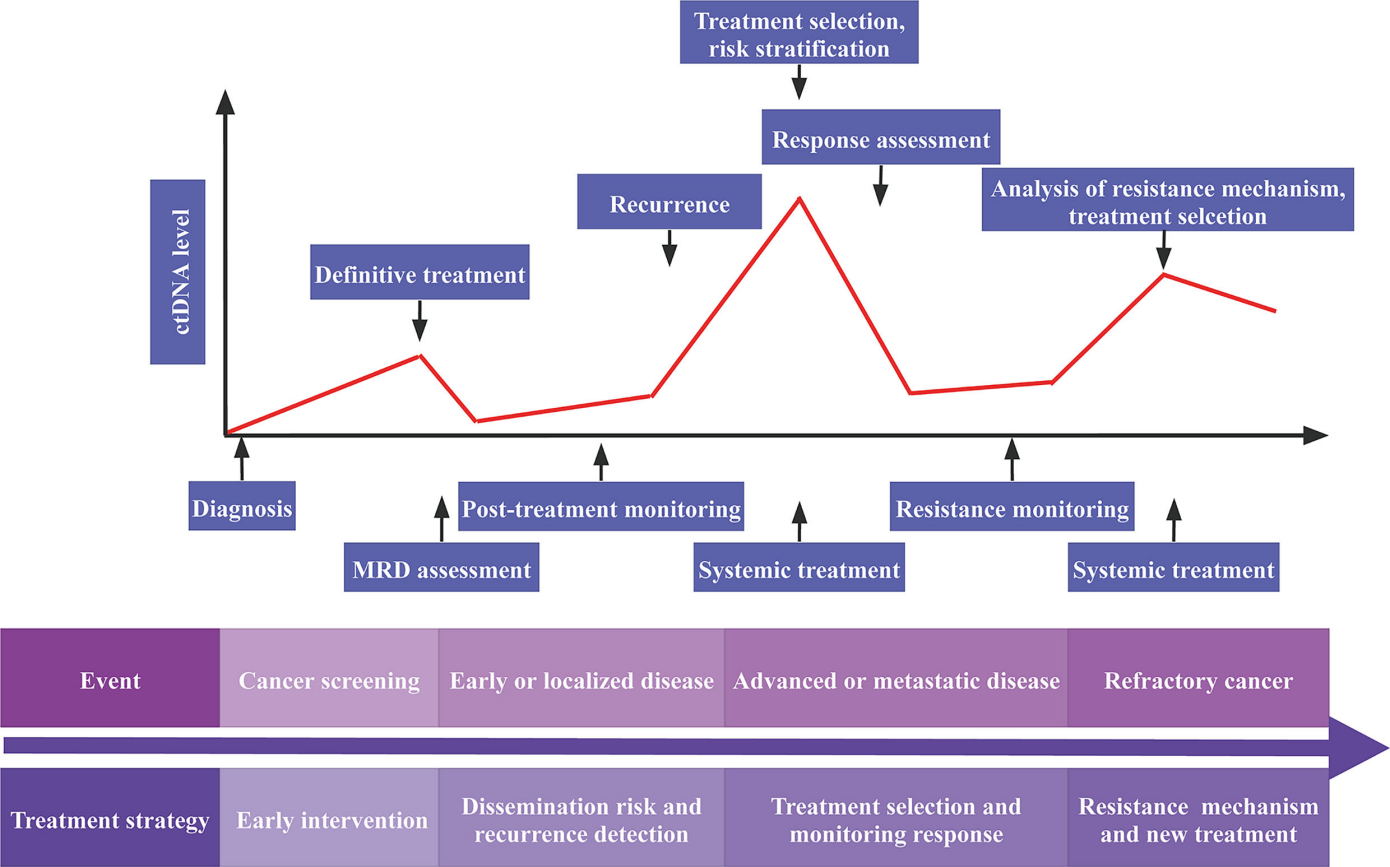
Potential clinical applications of ctDNA analysis. MRD, minimal residual disease; ctDNA, circulating tumor DNA.

Increasing evidence suggests that ctDNA may be used as a predictor of relapse risk (**Figure 2**). The presence of ctDNA in follow-up samples was linked to future recurrence in all major breast cancer subtypes, with ctDNA identified before relapse in 22 of 23 patients (95.7%) with extracranial distant metastatic relapse. TNBCs had the highest ctDNA levels upon diagnosis, suggesting rapid cell growth and turnover. Early identification of ctDNA before treatment raised the likelihood of recurrence in early-stage breast cancer (49). The phylogenetic ctDNA profiling is used for ctDNA-driven treatment research that monitors the subclonal nature of early-stage lung cancer recurrence and metastasis (50). Another study showed that nonmetastatic colorectal cancer patients with positive ctDNA had a recurrence incidence of 77%. Moreover, ctDNA positive patients had recurrence 3 months before radiologic or clinical evidence. With a median follow-up of 49 months, none of the 45 patients with negative ctDNA had a recurrence (51). The presence of ctDNA following cystectomy indicates the presence of residual cancer cells. After cystectomy, ctDNA was found in 17 individuals, 13 of whom had a recurrence. ctDNA-based recurrence detection outpaced radiographic imaging by up to a median of 96 days. Moreover, the dynamics of ctDNA throughout chemotherapy were related to disease recurrence (P =0.023) but not pathologic downstaging in ctDNA positive patients before or during therapy. The findings support that the feasibility of using ctDNA analysis for bladder cancer risk stratification, treatment monitoring, and early recurrence detection is feasible, and it offers a foundation for clinical trials evaluating early therapeutic approaches (52). Therefore, ctDNA may be utilized to identify early-stage cancer and predict recurrence in individuals with early-stage cancer.

### Monitoring Response Using ctDNA

There is mounting evidence that ctDNA analysis may be used to monitor the response to treatment intervention. After neoadjuvant chemotherapy, the presence of ctDNA was associated with a lower distant disease-free survival, disease-free survival, and overall survival in individuals with early-stage TNBC. Distant disease-free survival probability for ctDNA-positive individuals was 56% at 24 months, compared to 81% for ctDNA-negative patients (53). NCC-GP150 blood tumor mutational burden (bTMB) correlated well with WES matched tissue TMB (tTMB) (Spearman correlation = 0.62). A bTMB of 6 or higher was associated with improved progression-free survival and objective response rates in the anti-PD-1 and anti-PD-L1 therapy group, indicating that established NCC-GP150 with an optimized gene panel size and methodology was viable for bTMB estimation (54). Blood-based tumor mutational burden (bTMB) reliably identifies individuals who benefit from atezolizumab in second-line and higher NSCLC (55). 94% of patients with limited-stage (LS)-small-cell lung cancer (SCLC) and 100% of patients with extensive-stage (ES)-SCLC exhibited tumor-related alterations in their samples, including copy number alterations (CNAs) and somatic mutations. Targeted cfDNA sequencing reveals possible therapeutic targets in over 50% of SCLC patients using a simple cfDNA genomewide copy number method (56).

In advanced refractory CRC, patients with a plasma tumor mutation burden of 28 or more variations per megabase exhibited improved overall survival. Moreover, tumor mutation burden may be used to predict individuals with advanced refractory CRC who may benefit from durvalumab and tremelimumab (57). In the VIKTORY umbrella trail, ctDNA analysis revealed a strong link between high MET copy number and savolitinib response in patients with metastatic gastric cancer (58). At six weeks after immunotherapy, alterations in ctDNA levels suggested immunotherapeutic response and progression-free survival, and lower ctDNA levels were linked to better results. The results provide clues to the molecular characteristics associated with response to pembrolizumab in patients with metastatic gastric cancer (59). BRAF V600-mutant ctDNA identified in pre-treatment and on-treatment melanoma samples may be utilized as an independent indicator of clinical outcome in patients receiving dabrafenib or trametinib in combination with dabrafenib. In the COMbi-B cohort, the threshold of ctDNA was 64 copies per mL as a high or low risk of survival outcome, which was verified in the combi-B cohort. In the COMBI-d cohort, undetectable ctDNA at week four was associated with a prolonged progression-free and overall survival (60). Comprehensive ctDNA analysis reveals genetic variants that are clinically actionable in metastatic castration-resistant prostate cancer. Metastatic castration-resistant prostate cancer with TP53, BRCA2, or ATM mutations identified in plasma had substantially poorer outcomes (61). The analysis of ctDNA from patients with carcinoma of unknown primary (CUP) demonstrates the potential of the ctDNA method to provide tailored treatments to CUP patients (62).

ctDNA analysis can identify residual proliferating disease in adjuvant settings and estimate tumor burden in metastatic settings and is a stratification indicator for immune-checkpoint inhibition. Moreover, ctDNA testing for immunotherapy predictors such as mutations, tumor mutational burden, and microsatellite instability provides a noninvasive alternative to tumor biopsy sampling. Quantitative changes in ctDNA levels early in the disease course have also been shown to be a valuable technique for assessing immune-checkpoint inhibition response that may supplement conventional imaging approaches (63). In an analysis of immune checkpoint inhibition across a broad range of cancer types, elevated pretreatment variant allele frequencies (VAF) were linked to worse overall survival, implying that VAF plays a prognostic role in patient outcomes. On-treatment VAF decreases and decreased on-treatment VAF were related to prolonged progression-free survival and overall survival, indicating that on-treatment ctDNA dynamics are predictive of immune checkpoint blockade benefit. Moreover, the combination of pretreatment and on-treatment VAF using ctDNA can identify long-term responders and adjudicated benefit among individuals with initial radiologically stable disease in advanced cancers (64).

Taken together, ctDNA may aid in the precise treatment of cancer and may help monitor patients’ responses to treatment both during and after treatment.

### Resistance Mechanism Analysis Using ctDNA

ctDNA analysis can deepen the understanding of the mechanisms of drug resistance and provide more opportunities for precision medication for patients. The BENEFIT trial revealed that detecting EGFR mutations in ctDNA was an excellent method for identifying individuals who might benefit from gefitinib, and investigations of dynamic EGFR mutations and associated gene aberrances could help predict gefitinib resistance (65). BRCA reversion mutations are identified in 13% of platinum-resistant and 18% of platinum-refractory high-grade ovarian carcinoma pretreatment cfDNA and are associated with reduced therapeutic efficacy of rucaparib therapy. Besides, ctDNA analysis may identify several BRCA reversion mutations, indicating multiclonal heterogeneity in high-grade ovarian carcinoma (66). In almost all patients with mCRPC, clinically relevant genomic profiling of cfDNA was available, and it may offer significant insights on enzalutamide response and resistance (67). RAS and BRAF wild-type metastatic colorectal cancer patients responded to rechallenge with cetuximab and irinotecan in this phase 2 single-arm study. Only patients with RAS and BRAF wild-type ctDNA might benefit from the rechallenge, according to preplanned ctDNA profiling (68). CAPP-Seq analysis of ctDNA revealed that EGFR T790M mutation, MET amplification and ERBB2 amplification may lead to resistance to first- or second-generation EGFR-TKIs in NSCLC patients (69). Additional uses of ctDNA testing are being explored, including early identification of immunotherapy resistance and analysis of resistance pathways (63). In general, ctDNA provides a view into emerging mechanisms of resistance to targeted therapy or immunotherapy.

## Utilization of ctDNA for Minimal Residual Disease (MRD) Detection

**Figure 3:**
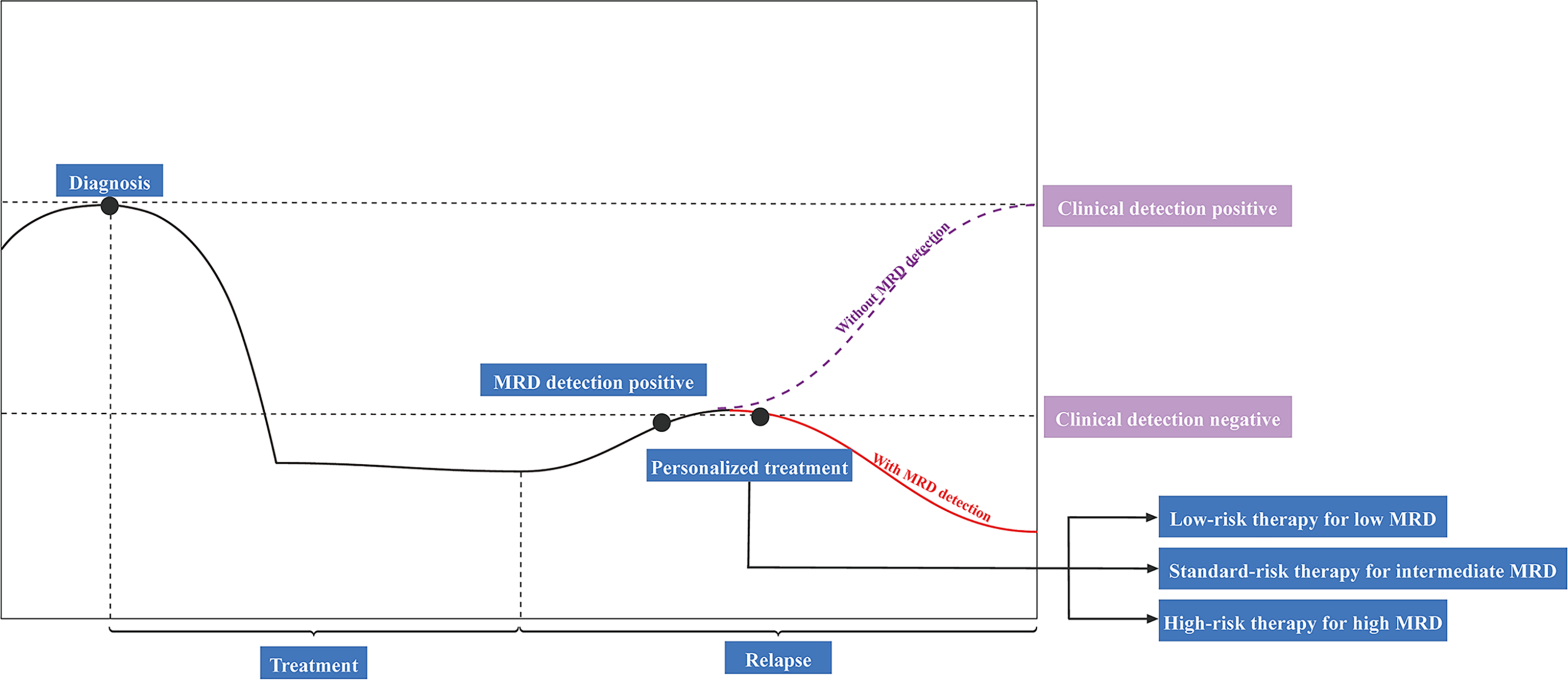
Potential clinical applications of ctDNA-based detection for minimal residual disease (MRD). ctDNA, circulating tumor DNA.

MRD refers to residual tumor cells or biomarkers in the body after local or systemic cancer treatment, and its activation promotes tumor metastasis and recurrence, which is described as minimal residual disease, measurable residual disease, and molecular residual disease. Because the number of remaining cancer cells is likely to be so tiny that they may not cause any signs or symptoms, and they may even be undetectable by conventional techniques. The commonly used MRD detection techniques include qPCR (quantitative PCR), ddPCR (digital PCR), NGS (next-generation sequencing). Among them, NGS, as an emerging MRD detection technique, is gaining increasing attention and clinical application. Early detection of tumor metastasis and recurrence is critical for extending survival because smaller tumors have a better prognosis. MRD is a significant prognostic indicator that may help predict recurrence. Recently, the use of ctDNA analysis to identify MRD in solid tumors after curative-intent therapy and before clinical or radiographic disease recurrence has demonstrated significant therapeutic promise (**Figure 3**). Besides, MRD identification by ctDNA analysis was associated with a poor prognosis in patients with a malignant tumor. In this study, we describe the significance of ctDNA analysis for guiding adjuvant therapy in lung, breast, and colon cancers.

### Lung Cancer

The TRACERx study showed that over 99% of MRD-negative patients did not relapse and that MRD predicted relapse before conventional imaging. The time gap between the rise in ctDNA levels after surgery and the clinical diagnosis of cancer recurrence offers an opportunity for clinical intervention (50). The DYNAMIC study is the first prospective research on exploring ctDNA dynamic alterations in primary lung cancer patients after surgery. After tumor excision, ctDNA decays quickly in individuals with surgical lung cancer. Three days following surgery may be used as a baseline for lung cancer postoperative monitoring and may help guide clinical decisions (70). Moreover, the DYNAMIC study discovered that the half-life of ctDNA in individuals with radical resected lung cancer was only 35 minutes and that detecting MRD on the third day following R0 resection may be utilized as a baseline for postoperative lung cancer monitoring (70, 71).

A retrospective study demonstrates that ctDNA analysis can reliably detect posttreatment MRD in patients with stage I-III lung cancer, detecting residual or recurrent disease earlier than standard radiologic imaging, and making tailored adjuvant therapy more accessible to patients at an early stage with the lowest disease burden. Freedom from progression (FFP) at 36 months after the MRD landmark was 0% in localized lung cancer patients with detectable ctDNA MRD and 93% in those with undetectable ctDNA MRD. The rate of MRD identification with single-mutation monitoring was 58%, considerably lower than the rate of 94% when all known variants were analyzed using cancer personalized profiling by deep sequencing (CAPP-seq), implying that monitoring multiple variants may improve MRD detection sensitivity in lung cancer (72). The assessment of MRD by ctDNA analysis predicts recurrence in early-stage lung cancer with excellent accuracy following therapy (72, 73). In another study that assessed the MRD in lung cancer patients using circulating single-molecule amplification and resequencing technology (cSMART), ctDNA status before surgery was a significant clinicopathological predictor for RFS and OS. ctDNA positive before surgery was associated with a 3.4- or 4.0-fold increased risk of recurrence or mortality, respectively. After surgery, the recurrence rate of ctDNA-positive patients was 63.3% (19/30). 89.5% of these patients who relapsed had detectable ctDNA within two weeks after surgery and were identified before imaging findings, with a median of 12.6 months (74).

In metastatic EGFR-mutant lung tumors, persistent EGFR-mutant ctDNA after six weeks of therapy was linked to early progression on osimertinib with bevacizumab and lower overall survival. In addition, persistently positive ctDNA may distinguish patients at risk for early progression of EGFR-TKIs and those who will benefit most from intensification therapy (75). In individuals who have had long-term responses to immune checkpoint inhibitors, ctDNA analysis can detect minimal residual disease and forecast the likelihood of disease progression. After a median of 26.7 months of immune checkpoint inhibitor therapy, ctDNA was tracked in 31 non–small cell lung cancer patients. 27 patients had undetectable ctDNA at the surveillance timepoint, and 93% (25/27) of them had not progressed. Besides, all four individuals with detectable ctDNA experienced disease progression. ctDNA monitoring may help to advance precision immunotherapy and provide more opportunities for early intervention in patients at high risk of disease progression (76).

Pre-treatment ctDNA and peripheral CD8 T levels are associated with durable clinical benefits from immune checkpoint inhibitors. ctDNA dynamics after a single infusion may help identify individuals who will obtain clinical benefits. Combining ctDNA with circulating immune cell profiling may determine patients who will benefit from treatment, and offer accurate, noninvasive, and early prediction of outcomes for NSCLC patients receiving immune checkpoint inhibitors (77). In the prospective INSPIRE study, all 12 patients whose ctDNA was cleared during pembrolizumab treatment had favorable clinical outcomes. With a median follow-up of 25.4 months after initial clearance, these 12 patients had sustained objective responses and 100% overall survival with a median of 25 months follow-up. For most patients with more than two ctDNA detections during ctDNA monitoring, increases in above-baseline ctDNA levels were linked to disease progression and poorer survival, with a median overall survival of 13.7 months. Besides, below-baseline ctDNA levels were associated with extended survival, with a median overall survival of 23.8 months. These findings indicated the therapeutic use of ctDNA-based monitoring in patients treated with immune checkpoint inhibitors (78).

### Breast Cancer

In a cohort of 55 early breast cancer patients undergoing neoadjuvant chemotherapy, identification of ctDNA following completing curative therapy accurately predicted metastatic recurrence. Mutation monitoring in serial samples increased sensitivity for recurrence prediction, with a median lead time of 7.9 months over clinical recurrence. Additionally, targeted capture sequencing of ctDNA could detect MRD-associated genetic events, and MRD detection more accurately predicted the genetic events associated with subsequent metastatic recurrence than primary cancer sequencing. Thus, mutation monitoring may be used to identify individuals with early-stage breast cancer who have an increased risk of recurrence. Subsequent adjuvant therapy may target genetic events identified in the MRD, partially overcoming the barrier posed by intratumor genetic heterogeneity (35).

Another clinical study revealed that identifying ctDNA at diagnosis, prior treatment in early-stage breast cancer was linked to relapse-free survival. When compared to clinical recurrence, ctDNA detection had a median lead time of 10.7 months and was associated with recurrence in all breast cancer subtypes, suggesting that molecular relapse detection may be used to guide adjuvant treatment (79). A recent study demonstrated that personalized ctDNA analysis utilizing targeted digital sequencing (TARDIS) could identify residual disease in stage I-III breast cancer patients with excellent accuracy after neoadjuvant treatment. TARDIS identified ctDNA in all patients with 0.11% median variant allele frequency (VAF) before therapy. Following neoadjuvant treatment, ctDNA levels were significantly lower in patients who achieved pathological complete response (pathCR) than in patients with residual disease. Additionally, individuals with pathCR had a substantial decrease in ctDNA levels after neoadjuvant treatment. These results indicate that it is possible to accurately evaluate the molecular response and residual disease utilizing ctDNA analysis during neoadjuvant treatment (80). In addition, a novel, ultrasensitive assay was established to monitor numbers of specific tumor mutations to identify MRD following therapy. Whole-exome sequencing was performed to identify mutations in tumor tissue. Subsequently, an individualized MRD assay was used to detect mutations in the cfDNA. This approach allows the accurate detection of MRD at tumor fractions up to 100-fold lower than the genomic equivalent (GE) limit. The presence of MRD at one year was significantly associated with distant recurrence. The median lead time between the initial positive sample and recurrence was 18.9 months (81).

Pretreatment biopsies were sequenced to evaluate the role of MRD in neoadjuvant therapy for breast cancer using a massive parallel sequencing (MPS) panel, which enabled the detection of mutations and their investigation in plasma using droplet digital PCR (ddPCR) and tagged targeted deep sequencing (tTDS) as complementary approaches. Over one deleterious mutation was identified using tTDS in all four relapsed patients, with an average lead time of six months before clinical recurrence. However, just one relapsed patient could be detected using ddPCR. The results indicated that tTDS is a non-invasive tool for MRD detection in breast cancer patients (82).

In the neoadjuvant I-SPY study, serial ctDNA analysis can be utilized to determine pathologic complete response and metastatic recurrence risk in neoadjuvant-treated breast cancer. Patients who sustained ctDNA positivity at three weeks after initiation of paclitaxel had a substantially greater likelihood of developing residual disease after neoadjuvant chemotherapy (83% non-pathologic complete response) than those who cleared ctDNA (52% non-pathologic complete response). Following neoadjuvant chemotherapy, 100% of patients (N=17) who achieved pathologic complete response were ctDNA negative. For those who failed to achieve pathologic complete response (N=43), 14% of patients with ctDNA positivity exhibited a substantially higher risk of metastatic recurrence. 86% of patients failed to achieve pathologic complete response and tested negative for ctDNA had a favorable prognosis. Insufficient ctDNA clearance was a strong predictor of worse response and metastatic recurrence. ctDNA clearance was linked to better survival even in individuals who failed to achieve pathologic complete response. Personalized ctDNA monitoring during high-risk early breast cancer neoadjuvant chemotherapy may help assess therapy response and survival (83).

Early ctDNA dynamics revealed a strong relationship between on-treatment ctDNA and shorter progression-free survival in PIK3CA mutant breast cancer treated with palbociclib, taselisib, and fulvestrant. During triplet therapy, sequencing of longitudinal plasma ctDNA revealed evidence of genetic evolution (84). The PALOMA-3 trial showed that the change in PIK3CA ctDNA levels after 15 days of palbociclib and fulvestrant therapy significantly predict progression-free survival. These findings indicated that early ctDNA dynamics might serve as a reliable biomarker for CDK4/6 inhibitors, with early ctDNA dynamics showing diverse responses to treatment of tumor subclones (85).

### Colorectal Cancer

In a prospective study, ctDNA-positive stage I to III CRC patients exhibited a seven-fold increased risk of recurrence at postoperative day thirty. Relapse was seventeen times more probable in ctDNA-positive individuals following adjuvant chemotherapy. After adjuvant chemotherapy, all seven ctDNA positive patients relapsed. According to post-treatment monitoring, adjuvant chemotherapy eliminated 30% of ctDNA-positive patients. ctDNA-positive patients had 40 times the risk of disease recurrence than ctDNA-negative individuals during monitoring following definitive treatment. Serial ctDNA analysis showed disease recurrence 16.5 months before conventional radiologic imaging. Overall, ctDNA analysis may improve postoperative CRC treatment by risk assessment, adjuvant chemotherapy monitoring, and early recurrence identification (86). Positive postoperative ctDNA results had poor outcomes despite adjuvant treatment, with a three-year recurrence-free interval of 47% vs. 76% in patients with negative postoperative ctDNA, indicating that ctDNA analysis might serve as a prognostic biomarker for recurrence risk and adjuvant therapy benefit in stage III colon cancer (87).

Customized next-generation sequencing (NGS) panels were used to detect mutations in localized colon cancer tissues. In addition, ddPCR was used to monitor a series of plasma samples for known and high-frequency ctDNA mutations. Identifying ctDNA in serial plasma samples was linked to worse disease-free survival (DFS). The capacity to detect MRD improved to 87.5% by monitoring more than two variants in plasma. The presence of ctDNA following treatment was linked to early recurrence in individuals who received adjuvant chemotherapy. ctDNA could be detected at follow-up before radiological relapse, with a median lead time of 11.5 months, indicating that tracking ctDNA mutations may aid in identifying recurrence and that identifying mutations in ctDNA occurring during or following adjuvant chemotherapy may aid in the detection of treatment resistance (88). ctDNA analysis after stage II colon cancer resection may demonstrate the presence of MRD, identify individuals at high risk of recurrence, and guide adjuvant treatment decisions. In a prospective cohort of 230 individuals with stage II colon cancer, parallel sequencing was performed to assess the capacity of ctDNA to identify MRD in 1046 plasma samples. Postoperative ctDNA was identified in 7.9% (14/178) of patients not treated with adjuvant chemotherapy. 78.6% (11/14) of these patients had radiologic recurrence at a median follow-up of 27 months. Only 9.8% (16/164) of the individuals with negative ctDNA had disease recurrence. The presence of ctDNA after completion of chemotherapy was linked to an inferior worse recurrence-free survival in those who underwent chemotherapy (89).

Recently, a plasma-only ctDNA assay that integrates genomic and epigenomic cancer signatures has been developed for tumor-uninformed MRD detection in postoperative colorectal cancer patients. Following completion of definitive treatment, 24% (17/70) of patients retained detectable ctDNA, and 88% (15/17) of these patients recurred. 24% (12/49) of the patients lacking detectable landmark ctDNA recurred. Sensitivity and specificity for landmark recurrence were 55.6% and 100%, respectively. The integration of longitudinal and surveillance analyses improved sensitivity to 69% and 91%, respectively. In comparison to genomic alterations alone, the integration of epigenomic signatures improved sensitivity by 25%–36%. The combination of epigenomic and genomic analyses improved sensitivity, indicating that plasma-only ctDNA MRD detection may be promising in clinical settings (90).

271 serial plasma samples were dynamically monitored with ctDNA during colorectal cancer patients with liver metastasis (CRLM) treatment to assess the impact of ctDNA on the prediction of adjuvant chemotherapy. Patients with a higher VAF level at their baseline ctDNA had a higher tumor burden, and reduced ctDNA levels during preoperative chemotherapy were associated with improved tumor response. The presence of ctDNA in patients after surgery and adjuvant chemotherapy was linked to a decreased recurrence-free survival (RFS). Patients with detectable ctDNA recurred after CRLM resection at a higher rate (79.4% vs. 41.7%) than those with undetectable ctDNA. Besides, recurrence rates were 77.3% for patients with detectable ctDNA following adjuvant chemotherapy and 40.7% for those with undetectable ctDNA. Patients with reduced ctDNA VAF had a 63.6% recurrence rate during adjuvant chemotherapy, compared to 92.3% for patients with elevated ctDNA VAF, indicating that dynamic ctDNA analysis in a post-adjuvant chemotherapy setting might be utilized to identify not only MRD but also to select the most appropriate individualized adjuvant treatment after CRLM resection (91).

## Other Tumors

Monitoring the copy number status of HER2 in ctDNA is beneficial for the therapeutic effect of patients with HER2-positive gastric cancer and identifying treatment options for patients whose HER2 status changes to positive following recurrence. Plasma samples collected during postoperative follow-up periods indicated that high plasma HER2 ratios were observed at recurrence in seven of the thirteen patients who were diagnosed as HER2-negative (92). MRD identified by ctDNA distinguished stage I-III gastric cancer individuals at high risk of postoperative relapse and enabled new adjuvant therapy studies to prolong survival in adjuvant treatment settings. In a prospective cohort study, all patients who had ctDNA detected immediately after surgery eventually experienced a relapse. Positive ctDNA at any timepoint during longitudinal postoperative follow-up was associated with worse disease-free survival and overall survival, with a median time of 6 months before radiographic recurrence (93).

After applying the white blood cells-filtering approach, the presence of ctDNA in the CRITICS trial predicts recurrence when assessed within nine weeks following preoperative therapy and following surgery in individuals suitable for multimodal therapy. After a median follow-up of 42 months, all 11 resectable gastric cancer patients with no identifiable tumor-specific alterations at the postoperative timepoint were alive and recurrence-free. Of the nine patients who had detectable tumor-specific alterations at the postoperative period, six patients experienced disease recurrence and died of metastatic disease. In addition, patients with identifiable tumor-specific alterations had a substantially lower median event-free survival (18.7 months vs. not reached) and a 21.8-fold higher risk of recurrence as well as a considerably shorter median overall survival (28.7 months vs. not reached) after surgery. Moreover, the time to recurrence was determined by ctDNA analysis at 1.4 months, 8.9 months earlier than clinical detection (94).

The prognostic and predictive value of ctDNA was investigated using ultra-deep sequencing in patients with locally advanced bladder cancer before and after cystectomy, as well as during chemotherapy. Pre-chemotherapy ctDNA presence was strongly prognostic at diagnosis. For surveillance after cystectomy, ctDNA positivity accurately predicted all patients with metastatic recurrence with 100% sensitivity and 98% specificity. The dynamics of ctDNA throughout chemotherapy were associated with recurrence in individuals with ctDNA positivity before or during chemotherapy (52). In urothelial carcinoma patients who are positive for ctDNA and are at a high risk of recurrence, adjuvant atezolizumab may be associated with better outcomes than observation. At the initiation of treatment, ctDNA assay revealed 37% of patients were positive for ctDNA and had a dismal prognosis. The atezolizumab arm outperformed the observation arm in terms of disease-free survival and overall survival. At week 6, the atezolizumab arm (18%) had a greater rate of ctDNA clearance than the observation arm (4%) (95).

Comprehensive ctDNA alteration profiles offer a reliable strategy for evaluating tumor burden, with high consistency with imaging findings. It was able to detect the presence of tumors before imaging for an average of 4.6 months, and it is superior to serum biomarkers, such as alpha-fetoprotein, alpha-fetoprotein-L3, and des-gamma-carboxy prothrombin. Moreover, it has the potential to accurately identify MRD in advance and forecast prognostic outcomes for relapse-free survival and overall survival. Comprehensive ctDNA alteration profiles may be used to evaluate prognostic risk and predict hepatocellular carcinoma occurrence (96).

## Challenges and Future Directions

An accurate understanding of the limitations of assay can effectively avoid making harmful decisions. Despite promising preliminary results, many obstacles exist to the widespread clinical application of ctDNA-based assay for treatment decision-making and tumor monitoring. In plasma, ctDNA levels tend to be variable and low, resulting in a variable detection threshold. In addition, negative ctDNA may be due to low copy number detection rather than the absence of ctDNA. The limited sensitivity of the ctDNA analysis is a critical challenge, particularly in patients with resected early-stage cancer, when plasma ctDNA levels are low. False negatives are inevitable due to the influence of biological variables such as mucinous histology, low DNA-shedding tumor, and hidden micrometastasis. NGS panels with a wide range of genomic or/and epigenetic alterations, larger sample volume, monitoring numerous mutations, serial testing, and fragment size analysis might enhance assay sensitivity. Besides, DNA fragments from the clonal hematopoiesis of indeterminate potential (CHIP) or non-neoplastic hematopoietic stem cells can cause false-positive ctDNA results, which can be reduced by utilizing advanced bioinformatics analysis or by comparing ctDNA sequencing with that of leukocytes and/or matched tumor tissues, but the optimal strategy is yet undetermined. A high-intensity cfDNA sequencing analysis method based on the combined analysis of cfDNA and white-blood-cell (WBC) gDNA enables *de novo* identification of tumor-derived alterations as well as interpretation of microsatellite instability, tumor mutational burden, mutational profiles, and the origins of somatic mutations found in cfDNA (97).

Integrating mutational information from peripheral blood cells (PBCs) is critical in liquid biopsy analysis to distinguish tumor-derived from clonal hematopoiesis (CH)-related mutations. Standard practice for NGS genomic analysis of cfDNA should include paired plasma-peripheral blood cell (PBC) sequencing to prevent findings from being misinterpreted (98). Another hurdle to overcome is the lack of uniformity among various ctDNA assays, which restricts the interpretation of presented results. The lack of standardization across ctDNA assays is another obstacle, which limits the understanding of available results. Discordance ctDNA findings are likely the consequence of several variables, including the time points of sample collection, sample collection process, storage procedure, library preparation process, unique molecular identifiers, variant calling, and targeted error correction. Normative methods for ctDNA collection, storage, and analysis are essential in ensuring the widespread utilization of ctDNA technology in regular clinical practice. Even though studies have provided convincing evidence supporting the role of ctDNA in the management of patients with resected early-stage cancer, these studies only included a small proportion of participants and lacked validation cohorts. Given the limited samples and observational findings, further large-scale randomized, controlled trials are required to verify and clarify the clinical usefulness of ctDNA in cancer.

The primary application of ctDNA assay in early-stage cancer treatment is its ability to identify MRD after primary tumor resection, thus enabling accurate risk assessment and adjuvant therapy. Adjuvant treatment may be avoided in the future for a significant proportion of ctDNA-negative individuals who are deemed high-risk. Moreover, ctDNA clearance may serve as an endpoint in adjuvant trials to assess the effectiveness of treatment, allowing for shorter follow-up times and smaller sample sizes. Besides, confirming adjuvant treatment duration based on ctDNA clearance will aid in reducing excessive toxicity. As a result, the ctDNA assay has enormous promise for speeding up the development of adjuvant therapies. Additional prospective studies will be conducted to evaluate the performance of the MRD assay. ctDNA surveillance during adjuvant therapy may aid in understanding the mechanisms of drug response and resistance, providing an opportunity for genome-based therapy before rapid disease progression. Neoadjuvant therapy is a rapidly developing approach for treating patients with early-stage cancer, and ctDNA may be an invaluable tool for monitoring tumor response in the neoadjuvant setting. It should be emphasized that excluding adjuvant treatment based on negative ctDNA assay is not appropriate, owing to the low degree of standardization of ctDNA testing procedures and the limitations of ctDNA testing technology.

Tumor-specific DNA methylation in plasma may be promising in cancer diagnosis, prognosis, and monitoring. With advances in molecular biology, detection technology, statistics, and machine learning, ctDNA methylation detection will make significant progress. Integrating DNA methylation analysis with genomic mutation detection may increase the sensitivity of detection. Incorporating various factors such as protein biomarkers, mutation-based, and epigenetic will get an accurate result. Genome-wide cell-free DNA fragmentation varies between cancer patients and healthy people. Fragmentation profiles of cfDNA in cancer patients seem to be caused by nucleosomal DNA mixes from both cancer cells and blood. DNA evaluation of fragments for early interception (DELFI) is a novel method that can identify a significant number of abnormalities in cfDNA *via* genome-wide evaluation of fragmentation patterns. By integrating DELFI with the detection of cfDNA sequence alterations, the sensitivity of detection was significantly improved. Since the fragmentation patterns appear to correlate with nucleosomal patterns, DELFI may help identify the tumor-derived ctDNA, which can be enhanced further utilizing clinical features, methylation alterations, and other diagnostic methods. Additionally, DELFI needs just a small amount of genome sequencing, implying that it has the potential to be widely applied to cancer screening and management (99).

A single-tube methylation-specific quantitative PCR method (mqMSP), using ten different methylation markers, was able to quantitatively assess plasma samples as low as 0.05% of tumor DNA. The mqMSP assay is a cost-efficient and easy-to-implement clinical monitoring method for colorectal cancer recurrence, which aids in patient management after surgery. 55% (N=20) of recurrence colon cancer had mqMSP positivity in the postoperative plasma samples, which was associated with worse recurrence-free survival. Among the 20 recurrence patients, 70% exhibited detectable ctDNA prior to recurrence, with a median lead time of 8.0 months earlier than radiologic imaging (100). The plasma cell-free DNA methylomes are a sensitive method to detect ctDNA in low-level input DNA. In a large number of plasma samples from a variety of tumor types, this method has shown its effectiveness in detecting and classifying using plasma cell-free DNA methylomes (29). Moreover, plasma cfDNA methylomes exhibit specific characteristics in detecting and discriminating common primary intracranial tumors, which share cell-of-origin lineages and are difficult to differentiate with standard-of-care imaging (101). Cell-free methylated DNA immunoprecipitation and high-throughput sequencing (cfMeDIP–seq) is a sensitive detection method that may identify cancers in the early stages. It can be used to accurately classify patients at all stages of renal cell carcinoma in plasma (the area under the receiver operating characteristic curve is 0.99), as wells as to identify renal cell carcinoma patients with urine cell-free DNA (the area under the receiver operating characteristic curve is 0.86), indicating that the utilization of plasma and urine cell-free DNA methylomes for the detection of renal cell carcinoma has the potential to revolutionize clinical practice (102).

Integrating methylated DNA immunoprecipitation with next-generation sequencing (MeDIP-seq) yields high-quality methylomes with typical resolutions of 100 to 300 bp at costs similar to those of capture-based methods. Moreover, the whole process, from DNA extraction to production of the MeDIP-seq library, may take around 3-5 days (103). Combining genetic and epigenetic characteristics of cfDNA can be used to distinguish between lung cancer and benign lung injury (BLN) plasma, indicating the potential of the multi-omics blood-based assay for non-invasive lung cancer management (104). Besides, ctDNA assays may have the potential to offer critical information on genomic heterogeneity. Adjuvant therapy may be guided by actionable mutations in clones that may vary from the primary tumor owing to clonal evolution or/and tumor heterogeneity. Surprisingly, there is evidence to substantiate the utility of ctDNA analysis in cancers of unknown origin (CUP) patients. Currently, the most data for ctDNA analysis mainly come from lung cancer, breast cancer, and colorectal cancer studies. The use of ctDNA will expand to a variety of tumor types, such as prostate cancer, and bladder cancer. It is anticipated that ctDNA will become increasingly extensively utilized and develop into a powerful tool for cancer diagnosis and treatment.

## Conclusion

As more evidence accumulates, it is becoming clear that ctDNA can be used as a biomarker for MRD detection and that it has the potential to aid in treatment decision-making. The advancement of ultra-sensitive ctDNA tests has the potential to improve cancer therapy. Moreover, ctDNA-based MRD detection may become an indispensable part of diagnosis and treatment. Using ctDNA detection techniques to evaluate MRD after therapeutic surgery may radically alter the course of adjuvant therapy for non-metastatic cancer. Serial postoperative ctDNA analysis may provide more accurate risk stratification for recurrence in addition to pathological staging. Moreover, postoperative ctDNA analysis may be used to adjust the intensity and duration of adjuvant treatment depending on the ctDNA findings. ctDNA monitoring can predict the effectiveness of adjuvant treatment and enhance the efficiency of adjuvant therapy trials. More studies are required to validate the clinical effectiveness of ctDNA and further enhance the sensitivity of ctDNA analysis. ctDNA assay standards should be established to ensure the repeatability of the results. Overall, the application of ctDNA-based MRD analysis is of great benefit in providing clinical decision support and enhancing patient survival outcomes in the era of precision medicine.

## Author Contributions

All authors listed have made a substantial, direct, and intellectual contribution to the work, and approved it for publication.

## Funding

This work was supported by the National Natural Science Foundation of China (81660755); and the Science and Technology Project of Shenzhen of China (JCYJ20170307160524377 and JCYJ20190808162605484).

## Conflict of Interest

The authors declare that the research was conducted in the absence of any commercial or financial relationships that could be construed as a potential conflict of interest.

## Publisher’s Note

All claims expressed in this article are solely those of the authors and do not necessarily represent those of their affiliated organizations, or those of the publisher, the editors and the reviewers. Any product that may be evaluated in this article, or claim that may be made by its manufacturer, is not guaranteed or endorsed by the publisher.

## References

[1] Bohers E Viailly PJ Jardin F. cfDNA Sequencing: Technological Approaches and Bioinformatic Issues. Pharmaceuticals (Basel) (2021) 14(6):596. doi: 10.3390/ph14060596 34205827 PMC8234829

[2] Chin RI Chen K Usmani A Chua C Harris PK Binkley MS. Detection of Solid Tumor Molecular Residual Disease (MRD) Using Circulating Tumor DNA (ctDNA). Mol Diagn Ther (2019) 23(3):311–31. doi: 10.1007/s40291-019-00390-5 PMC6561896 30941670

[3] Bronkhorst AJ Ungerer V Holdenrieder S. The Emerging Role of Cell-Free DNA as a Molecular Marker for Cancer Management. Biomol Detect Quantif (2019) 17:100087. doi: 10.1016/j.bdq.2019.100087 30923679 PMC6425120

[4] Coakley M Garcia-Murillas I Turner NC. Molecular Residual Disease and Adjuvant Trial Design in Solid Tumors. Clin Cancer Res (2019) 25(20):6026–34. doi: 10.1158/1078-0432.CCR-19-0152 31088829

[5] Guibert N Pradines A Favre G Mazieres J. Current and Future Applications of Liquid Biopsy in Nonsmall Cell Lung Cancer From Early to Advanced Stages. Eur Respir Rev (2020) 29(155):190052. doi: 10.1183/16000617.0052-2019 32051167 PMC9488537

[6] Herberts C Wyatt AW. Technical and Biological Constraints on ctDNA-Based Genotyping. Trends Cancer (2021) 7(11):995–1009. doi: 10.1016/j.trecan.2021.06.001 34219051

[7] Little S. Amplification-Refractory Mutation System (ARMS) Analysis of Point Mutations. Curr Protoc Hum Genet (2001) Chapter 9:Unit 9 8. doi: 10.1002/0471142905.hg0908s07 18428319

[8] Postel M Roosen A Laurent-Puig P Taly V Wang-Renault SF. Droplet-Based Digital PCR and Next Generation Sequencing for Monitoring Circulating Tumor DNA: A Cancer Diagnostic Perspective. Expert Rev Mol Diagn (2018) 18(1):7–17. doi: 10.1080/14737159.2018.1400384 29115895

[9] Valpione S Campana L. Detection of Circulating Tumor DNA (ctDNA) by Digital Droplet Polymerase Chain Reaction (Dd-PCR) in Liquid Biopsies. Methods Enzymol (2019) 629:1–15. doi: 10.1016/bs.mie.2019.08.002 31727235

[10] O’Leary B Hrebien S Beaney M Fribbens C Garcia-Murillas I Jiang J. Comparison of BEAMing and Droplet Digital PCR for Circulating Tumor DNA Analysis. Clin Chem (2019) 65(11):1405–13. doi: 10.1373/clinchem.2019.305805 31551314

[11] Garcia-Foncillas J Alba E Aranda E Diaz-Rubio E Lopez-Lopez R Tabernero J. Incorporating BEAMing Technology as a Liquid Biopsy Into Clinical Practice for the Management of Colorectal Cancer Patients: An Expert Taskforce Review. Ann Oncol (2017) 28(12):2943–9. doi: 10.1093/annonc/mdx501 PMC5834030 28945877

[12] Gale D Lawson ARJ Howarth K Madi M Durham B Smalley S. Development of a Highly Sensitive Liquid Biopsy Platform to Detect Clinically-Relevant Cancer Mutations at Low Allele Fractions in Cell-Free DNA. PloS One (2018) 13(3):e0194630. doi: 10.1371/journal.pone.0194630 29547634 PMC5856404

[13] Forshew T Murtaza M Parkinson C Gale D Tsui DW Kaper F. Noninvasive Identification and Monitoring of Cancer Mutations by Targeted Deep Sequencing of Plasma DNA. Sci Transl Med (2012) 4(136):136ra68. doi: 10.1126/scitranslmed.3003726 22649089

[14] Fredebohm J Mehnert DH Lober AK Holtrup F van Rahden V Angenendt P. Detection and Quantification of KIT Mutations in ctDNA by Plasma Safe-SeqS. Adv Exp Med Biol (2016) 924:187–9. doi: 10.1007/978-3-319-42044-8_34 27753042

[15] Tie J Wang Y Cohen J Li L Hong W Christie M. Circulating Tumor DNA Dynamics and Recurrence Risk in Patients Undergoing Curative Intent Resection of Colorectal Cancer Liver Metastases: A Prospective Cohort Study. PloS Med (2021) 18(5):e1003620. doi: 10.1371/journal.pmed.1003620 33939694 PMC8128260

[16] Chabon JJ Hamilton EG Kurtz DM Esfahani MS Moding EJ Stehr H. Integrating Genomic Features for Non-Invasive Early Lung Cancer Detection. Nature (2020) 580(7802):245–51. doi: 10.1038/s41586-020-2140-0 PMC8230734 32269342

[17] Bratman SV Newman AM Alizadeh AA Diehn M. Potential Clinical Utility of Ultrasensitive Circulating Tumor DNA Detection With CAPP-Seq. Expert Rev Mol Diagn (2015) 15(6):715–9. doi: 10.1586/14737159.2015.1019476 PMC5052032 25773944

[18] Phallen J Sausen M Adleff V Leal A Hruban C White J. Direct Detection of Early-Stage Cancers Using Circulating Tumor DNA. Sci Transl Med (2017) 9(403):eaan2415. doi: 10.1126/scitranslmed.aan2415 28814544 PMC6714979

[19] Belic J Koch M Ulz P Auer M Gerhalter T Mohan S. Rapid Identification of Plasma DNA Samples With Increased ctDNA Levels by a Modified FAST-SeqS Approach. Clin Chem (2015) 61(6):838–49. doi: 10.1373/clinchem.2014.234286 25896989

[20] Belic J Koch M Ulz P Auer M Gerhalter T Mohan S. Mfast-SeqS as a Monitoring and Pre-Screening Tool for Tumor-Specific Aneuploidy in Plasma DNA. Adv Exp Med Biol (2016) 924:147–55. doi: 10.1007/978-3-319-42044-8_28 27753036

[21] Imperial R Nazer M Ahmed Z Kam AE Pluard TJ Bahaj W. Matched Whole-Genome Sequencing (WGS) and Whole-Exome Sequencing (WES) of Tumor Tissue With Circulating Tumor DNA (ctDNA) Analysis: Complementary Modalities in Clinical Practice. Cancers (Basel) (2019) 11(9):1399. doi: 10.3390/cancers11091399 PMC6770276 31546879

[22] Li H Jing C Wu J Ni J Sha H Xu X. Circulating Tumor DNA Detection: A Potential Tool for Colorectal Cancer Management. Oncol Lett (2019) 17(2):1409–16. doi: 10.3892/ol.2018.9794 PMC6341840 30675194

[23] Elazezy M Joosse SA. Techniques of Using Circulating Tumor DNA as a Liquid Biopsy Component in Cancer Management. Comput Struct Biotechnol J (2018) 16:370–8. doi: 10.1016/j.csbj.2018.10.002 PMC6197739 30364656

[24] Li C He Q Liang H Cheng B Li J Xiong S. Diagnostic Accuracy of Droplet Digital PCR and Amplification Refractory Mutation System PCR for Detecting EGFR Mutation in Cell-Free DNA of Lung Cancer: A Meta-Analysis. Front Oncol (2020) 10:290. doi: 10.3389/fonc.2020.00290 32195189 PMC7063461

[25] Janku F Huang HJ Fujii T Shelton DN Madwani K Fu S. Multiplex KRASG12/G13 Mutation Testing of Unamplified Cell-Free DNA From the Plasma of Patients With Advanced Cancers Using Droplet Digital Polymerase Chain Reaction. Ann Oncol (2017) 28(3):642–50. doi: 10.1093/annonc/mdw670 PMC5834133 27993791

[26] Kerachian MA Poudineh A Thiery JP. Cell Free Circulating Tumor Nucleic Acids, a Revolution in Personalized Cancer Medicine. Crit Rev Oncol Hematol (2019) 144:102827. doi: 10.1016/j.critrevonc.2019.102827 31715326

[27] Franczak C Filhine-Tresarrieu P Gilson P Merlin JL Au L Harle A. Technical Considerations for Circulating Tumor DNA Detection in Oncology. Expert Rev Mol Diagn (2019) 19(2):121–35. doi: 10.1080/14737159.2019.1568873 30648442

[28] Newman AM Lovejoy AF Klass DM Kurtz DM Chabon JJ Scherer F. Integrated Digital Error Suppression for Improved Detection of Circulating Tumor DNA. Nat Biotechnol (2016) 34(5):547–55. doi: 10.1038/nbt.3520 PMC4907374 27018799

[29] Shen SY Singhania R Fehringer G Chakravarthy A Roehrl MHA Chadwick D. Sensitive Tumour Detection and Classification Using Plasma Cell-Free DNA Methylomes. Nature (2018) 563(7732):579–83. doi: 10.1038/s41586-018-0703-0 30429608

[30] Mandel P Metais P. Nuclear Acids In Human Blood Plasma. C R Seances Soc Biol Fil (1948) 142(3-4):241–3. 18875018

[31] Leon SA Shapiro B Sklaroff DM Yaros MJ. Free DNA in the Serum of Cancer Patients and the Effect of Therapy. Cancer Res (1977) 37(3):646–50. 837366

[32] Sorenson GD Pribish DM Valone FH Memoli VA Bzik DJ Yao SL. Soluble Normal and Mutated DNA Sequences From Single-Copy Genes in Human Blood. Cancer Epidemiol Biomarkers Prev (1994) 3(1):67–71. 8118388

[33] Diehl F Schmidt K Choti MA Romans K Goodman S Li M. Circulating Mutant DNA to Assess Tumor Dynamics. Nat Med (2008) 14(9):985–90. doi: 10.1038/nm.1789 PMC2820391 18670422

[34] Dawson SJ Tsui DW Murtaza M Biggs H Rueda OM Chin SF. Analysis of Circulating Tumor DNA to Monitor Metastatic Breast Cancer. N Engl J Med (2013) 368(13):1199–209. doi: 10.1056/NEJMoa1213261 23484797

[35] Garcia-Murillas I Schiavon G Weigelt B Ng C Hrebien S Cutts RJ. Mutation Tracking in Circulating Tumor DNA Predicts Relapse in Early Breast Cancer. Sci Transl Med (2015) 7(302):302ra133. doi: 10.1126/scitranslmed.aab0021 26311728

[36] Alix-Panabieres C Pantel K. Liquid Biopsy: From Discovery to Clinical Application. Cancer Discov (2021) 11(4):858–73. doi: 10.1158/2159-8290.CD-20-1311 33811121

[37] Osumi H Shinozaki E Yamaguchi K Zembutsu H. Clinical Utility of Circulating Tumor DNA for Colorectal Cancer. Cancer Sci (2019) 110(4):1148–55. doi: 10.1111/cas.13972 PMC6447957 30742729

[38] Bailey C Black JRM Reading JL Litchfield K Turajlic S McGranahan N. Tracking Cancer Evolution Through the Disease Course. Cancer Discov (2021) 11(4):916–32. doi: 10.1158/2159-8290.CD-20-1559 PMC7611362 33811124

[39] Lin LH Allison DHR Feng Y Jour G Park K Zhou F. Comparison of Solid Tissue Sequencing and Liquid Biopsy Accuracy in Identification of Clinically Relevant Gene Mutations and Rearrangements in Lung Adenocarcinomas. Mod Pathol (2021). doi: 10.1038/s41379-021-00880-0 34362997

[40] Aggarwal C Thompson JC Black TA Katz SI Fan R Yee SS. Clinical Implications of Plasma-Based Genotyping With the Delivery of Personalized Therapy in Metastatic Non-Small Cell Lung Cancer. JAMA Oncol (2019) 5(2):173–80. doi: 10.1001/jamaoncol.2018.4305 PMC6396811 30325992

[41] Rosell R Karachaliou N. Lung Cancer: Using ctDNA to Track EGFR and KRAS Mutations in Advanced-Stage Disease. Nat Rev Clin Oncol (2016) 13(7):401–2. doi: 10.1038/nrclinonc.2016.83 27245284

[42] Sidaway P. Prostate Cancer: Mutations in ctDNA Reflect Features of Metastatic Disease. Nat Rev Clin Oncol (2017) 14(9):526. doi: 10.1038/nrclinonc.2017.111 28719582

[43] Schweizer MT Sivakumar S Tukachinsky H Coleman I De Sarkar N Yu EY. Concordance of DNA Repair Gene Mutations in Paired Primary Prostate Cancer Samples and Metastatic Tissue or Cell-Free DNA. JAMA Oncol (2021) 7(9):1378–82. doi: 10.1001/jamaoncol.2021.2350 PMC8446811 34086042

[44] Turner NC Kingston B Kilburn LS Kernaghan S Wardley AM Macpherson IR. Circulating Tumour DNA Analysis to Direct Therapy in Advanced Breast Cancer (plasmaMATCH): A Multicentre, Multicohort, Phase 2a, Platform Trial. Lancet Oncol (2020) 21(10):1296–308. doi: 10.1016/S1470-2045(20)30444-7 PMC7599319 32919527

[45] Parsons HA Beaver JA Cimino-Mathews A Ali SM Axilbund J Chu D. Individualized Molecular Analyses Guide Efforts (IMAGE): A Prospective Study of Molecular Profiling of Tissue and Blood in Metastatic Triple-Negative Breast Cancer. Clin Cancer Res (2017) 23(2):379–86. doi: 10.1158/1078-0432.CCR-16-1543 PMC5241251 27489289

[46] Stover DG Parsons HA Ha G Freeman SS Barry WT Guo H. Association of Cell-Free DNA Tumor Fraction and Somatic Copy Number Alterations With Survival in Metastatic Triple-Negative Breast Cancer. J Clin Oncol (2018) 36(6):543–53. doi: 10.1200/JCO.2017.76.0033 PMC5815405 29298117

[47] Heitzer E Ulz P Belic J Gutschi S Quehenberger F Fischereder K. Tumor-Associated Copy Number Changes in the Circulation of Patients With Prostate Cancer Identified Through Whole-Genome Sequencing. Genome Med (2013) 5(4):30. doi: 10.1186/gm434 23561577 PMC3707016

[48] Nakamura Y Taniguchi H Ikeda M Bando H Kato K Morizane C. Clinical Utility of Circulating Tumor DNA Sequencing in Advanced Gastrointestinal Cancer: SCRUM-Japan GI-SCREEN and GOZILA Studies. Nat Med (2020) 26(12):1859–64. doi: 10.1038/s41591-020-1063-5 33020649

[49] Karthikeyan S Park BH. Circulating Tumor DNA as a Marker for Disease Relapse in Early-Stage Breast Cancer-Bad Blood. JAMA Oncol (2019) 5(10):1479–80. doi: 10.1001/jamaoncol.2019.2047 31369044

[50] Abbosh C Birkbak NJ Wilson GA Jamal-Hanjani M Constantin T Salari R. Phylogenetic ctDNA Analysis Depicts Early-Stage Lung Cancer Evolution. Nature (2017) 545(7655):446–51. doi: 10.1038/nature22364 PMC5812436 28445469

[51] Wang Y Li L Cohen JD Kinde I Ptak J Popoli M. Prognostic Potential of Circulating Tumor DNA Measurement in Postoperative Surveillance of Nonmetastatic Colorectal Cancer. JAMA Oncol (2019) 5(8):1118–23. doi: 10.1001/jamaoncol.2019.0512 PMC6512291 31070668

[52] Christensen E Birkenkamp-Demtroder K Sethi H Shchegrova S Salari R Nordentoft I. Early Detection of Metastatic Relapse and Monitoring of Therapeutic Efficacy by Ultra-Deep Sequencing of Plasma Cell-Free DNA in Patients With Urothelial Bladder Carcinoma. J Clin Oncol (2019) 37(18):1547–57. doi: 10.1200/JCO.18.02052 31059311

[53] Radovich M Jiang G Hancock BA Chitambar C Nanda R Falkson C. Association of Circulating Tumor DNA and Circulating Tumor Cells After Neoadjuvant Chemotherapy With Disease Recurrence in Patients With Triple-Negative Breast Cancer: Preplanned Secondary Analysis of the BRE12-158 Randomized Clinical Trial. JAMA Oncol (2020) 6(9):1410–5. doi: 10.1001/jamaoncol.2020.2295 PMC7349081 32644110

[54] Wang Z Duan J Cai S Han M Dong H Zhao J. Assessment of Blood Tumor Mutational Burden as a Potential Biomarker for Immunotherapy in Patients With Non-Small Cell Lung Cancer With Use of a Next-Generation Sequencing Cancer Gene Panel. JAMA Oncol (2019) 5(5):696–702. doi: 10.1001/jamaoncol.2018.7098 30816954 PMC6512308

[55] Gandara DR Paul SM Kowanetz M Schleifman E Zou W Li Y. Blood-Based Tumor Mutational Burden as a Predictor of Clinical Benefit in Non-Small-Cell Lung Cancer Patients Treated With Atezolizumab. Nat Med (2018) 24(9):1441–8. doi: 10.1038/s41591-018-0134-3 30082870

[56] Mohan S Foy V Ayub M Leong HS Schofield P Sahoo S. Profiling of Circulating Free DNA Using Targeted and Genome-Wide Sequencing in Patients With SCLC. J Thorac Oncol (2020) 15(2):216–30. doi: 10.1016/j.jtho.2019.10.007 PMC7001105 31629061

[57] Chen EX Jonker DJ Loree JM Kennecke HF Berry SR Couture F. Effect of Combined Immune Checkpoint Inhibition vs Best Supportive Care Alone in Patients With Advanced Colorectal Cancer: The Canadian Cancer Trials Group CO.26 Study. JAMA Oncol (2020) 6(6):831–8. doi: 10.1001/jamaoncol.2020.0910 PMC7206536 32379280

[58] Lee J Kim ST Kim K Lee H Kozarewa I Mortimer PGS. Tumor Genomic Profiling Guides Patients With Metastatic Gastric Cancer to Targeted Treatment: The VIKTORY Umbrella Trial. Cancer Discov (2019) 9(10):1388–405. doi: 10.1158/2159-8290.CD-19-0442 31315834

[59] Kim ST Cristescu R Bass AJ Kim KM Odegaard JI Kim K. Comprehensive Molecular Characterization of Clinical Responses to PD-1 Inhibition in Metastatic Gastric Cancer. Nat Med (2018) 24(9):1449–58. doi: 10.1038/s41591-018-0101-z 30013197

[60] Syeda MM Wiggins JM Corless BC Long GV Flaherty KT Schadendorf D. Circulating Tumour DNA in Patients With Advanced Melanoma Treated With Dabrafenib or Dabrafenib Plus Trametinib: A Clinical Validation Study. Lancet Oncol (2021) 22(3):370–80. doi: 10.1016/S1470-2045(20)30726-9 PMC8034833 33587894

[61] Jayaram A Wetterskog D Attard G. Plasma DNA and Metastatic Castration-Resistant Prostate Cancer: The Odyssey to a Clinical Biomarker Test. Cancer Discov (2018) 8(4):392–4. doi: 10.1158/2159-8290.CD-18-0124 29610288

[62] Sidaway P. Targeted Therapy: ctDNA Identified in Patients With CUP. Nat Rev Clin Oncol (2017) 14(9):524. doi: 10.1038/nrclinonc.2017.105 28695912

[63] Cabel L Proudhon C Romano E Girard N Lantz O Stern MH. Clinical Potential of Circulating Tumour DNA in Patients Receiving Anticancer Immunotherapy. Nat Rev Clin Oncol (2018) 15(10):639–50. doi: 10.1038/s41571-018-0074-3 30050094

[64] Zhang Q Luo J Wu S Si H Gao C Xu W. Prognostic and Predictive Impact of Circulating Tumor DNA in Patients With Advanced Cancers Treated With Immune Checkpoint Blockade. Cancer Discov (2020) 10(12):1842–53. doi: 10.1158/2159-8290.CD-20-0047 PMC8358981 32816849

[65] Wang Z Cheng Y An T Gao H Wang K Zhou Q. Detection of EGFR Mutations in Plasma Circulating Tumour DNA as a Selection Criterion for First-Line Gefitinib Treatment in Patients With Advanced Lung Adenocarcinoma (BENEFIT): A Phase 2, Single-Arm, Multicentre Clinical Trial. Lancet Respir Med (2018) 6(9):681–90. doi: 10.1016/S2213-2600(18)30264-9 30017884

[66] Lin KK Harrell MI Oza AM Oaknin A Ray-Coquard I Tinker AV. BRCA Reversion Mutations in Circulating Tumor DNA Predict Primary and Acquired Resistance to the PARP Inhibitor Rucaparib in High-Grade Ovarian Carcinoma. Cancer Discov (2019) 9(2):210–9. doi: 10.1158/2159-8290.CD-18-0715 30425037

[67] Wyatt AW Azad AA Volik SV Annala M Beja K McConeghy B. Genomic Alterations in Cell-Free DNA and Enzalutamide Resistance in Castration-Resistant Prostate Cancer. JAMA Oncol (2016) 2(12):1598–606. doi: 10.1001/jamaoncol.2016.0494 PMC5097690 27148695

[68] Cremolini C Rossini D Dell’Aquila E Lonardi S Conca E Del Re M. Rechallenge for Patients With RAS and BRAF Wild-Type Metastatic Colorectal Cancer With Acquired Resistance to First-Line Cetuximab and Irinotecan: A Phase 2 Single-Arm Clinical Trial. JAMA Oncol (2019) 5(3):343–50. doi: 10.1001/jamaoncol.2018.5080 PMC6439839 30476968

[69] Otsubo K Sakai K Takeshita M Harada D Azuma K Ota K. Genetic Profiling of Non-Small Cell Lung Cancer at Development of Resistance to First- or Second-Generation EGFR-TKIs by CAPP-Seq Analysis of Circulating Tumor DNA. Oncologist (2019) 24(8):1022–6. doi: 10.1634/theoncologist.2019-0101 PMC6693714 31023862

[70] Chen K Zhao H Shi Y Yang F Wang LT Kang G. Perioperative Dynamic Changes in Circulating Tumor DNA in Patients With Lung Cancer (DYNAMIC). Clin Cancer Res (2019) 25(23):7058–67. doi: 10.1158/1078-0432.CCR-19-1213 31439586

[71] Killock D. DYNAMIC Insights Into MRD Responses Early After Resection of NSCLC. Nat Rev Clin Oncol (2019) 16(11):661. doi: 10.1038/s41571-019-0274-5 31485031

[72] Chaudhuri AA Chabon JJ Lovejoy AF Newman AM Stehr H Azad TD. Early Detection of Molecular Residual Disease in Localized Lung Cancer by Circulating Tumor DNA Profiling. Cancer Discov (2017) 7(12):1394–403. doi: 10.1158/2159-8290.CD-17-0716 PMC5895851 28899864

[73] Comino-Mendez I Turner N. Predicting Relapse With Circulating Tumor DNA Analysis in Lung Cancer. Cancer Discov (2017) 7(12):1368–70. doi: 10.1158/2159-8290.CD-17-1086 29208774

[74] Peng M Huang Q Yin W Tan S Chen C Liu W. Circulating Tumor DNA as a Prognostic Biomarker in Localized Non-Small Cell Lung Cancer. Front Oncol (2020) 10:561598. doi: 10.3389/fonc.2020.561598 33042842 PMC7523087

[75] Yu HA Schoenfeld AJ Makhnin A Kim R Rizvi H Tsui D. Effect of Osimertinib and Bevacizumab on Progression-Free Survival for Patients With Metastatic EGFR-Mutant Lung Cancers: A Phase 1/2 Single-Group Open-Label Trial. JAMA Oncol (2020) 6(7):1048–54. doi: 10.1001/jamaoncol.2020.1260 PMC7256866 32463456

[76] Hellmann MD Nabet BY Rizvi H Chaudhuri AA Wells DK Dunphy MPS. Circulating Tumor DNA Analysis to Assess Risk of Progression After Long-Term Response to PD-(L)1 Blockade in NSCLC. Clin Cancer Res (2020) 26(12):2849–58. doi: 10.1158/1078-0432.CCR-19-3418 PMC7299781 32046999

[77] Nabet BY Esfahani MS Moding EJ Hamilton EG Chabon JJ Rizvi H. Noninvasive Early Identification of Therapeutic Benefit From Immune Checkpoint Inhibition. Cell (2020) 183(2):363–76 e13. doi: 10.1016/j.cell.2020.09.001 33007267 PMC7572899

[78] Bratman SV Yang SYC Iafolla MAJ Liu Z Hansen AR Bedard PL. Personalized Circulating Tumor DNA Analysis as a Predictive Biomarker in Solid Tumor Patients Treated With Pembrolizumab. Nat Cancer (2020) 1(9):873–81. doi: 10.1038/s43018-020-0096-5 35121950

[79] Garcia-Murillas I Chopra N Comino-Mendez I Beaney M Tovey H Cutts RJ. Assessment of Molecular Relapse Detection in Early-Stage Breast Cancer. JAMA Oncol (2019) 5(10):1473–8. doi: 10.1001/jamaoncol.2019.1838 PMC6681568 31369045

[80] McDonald BR Contente-Cuomo T Sammut SJ Odenheimer-Bergman A Ernst B Perdigones N. Personalized Circulating Tumor DNA Analysis to Detect Residual Disease After Neoadjuvant Therapy in Breast Cancer. Sci Transl Med (2019) 11(504):eaax7392. doi: 10.1126/scitranslmed.aax7392 31391323 PMC7236617

[81] Parsons HA Rhoades J Reed SC Gydush G Ram P Exman P. Sensitive Detection of Minimal Residual Disease in Patients Treated for Early-Stage Breast Cancer. Clin Cancer Res (2020) 26(11):2556–64. doi: 10.1158/1078-0432.CCR-19-3005 PMC7654718 32170028

[82] Cirmena G Garuti A De Mariano M Coco S Ferrando L Isnaldi E. Circulating Tumor DNA Using Tagged Targeted Deep Sequencing to Assess Minimal Residual Disease in Breast Cancer Patients Undergoing Neoadjuvant Chemotherapy. J Oncol (2020) 2020:8132507. doi: 10.1155/2020/8132507 32377196 PMC7196957

[83] Magbanua MJM Swigart LB Wu HT Hirst GL Yau C Wolf DM. Circulating Tumor DNA in Neoadjuvant-Treated Breast Cancer Reflects Response and Survival. Ann Oncol (2021) 32(2):229–39. doi: 10.1016/j.annonc.2020.11.007 PMC9348585 33232761

[84] Pascual J Lim JSJ Macpherson IR Armstrong AC Ring A Okines AFC. Triplet Therapy With Palbociclib, Taselisib, and Fulvestrant in PIK3CA-Mutant Breast Cancer and Doublet Palbociclib and Taselisib in Pathway-Mutant Solid Cancers. Cancer Discov (2021) 11(1):92–107. doi: 10.1158/2159-8290.CD-20-0553 32958578

[85] O’Leary B Hrebien S Morden JP Beaney M Fribbens C Huang X. Early Circulating Tumor DNA Dynamics and Clonal Selection With Palbociclib and Fulvestrant for Breast Cancer. Nat Commun (2018) 9(1):896. doi: 10.1038/s41467-018-03215-x 29497091 PMC5832789

[86] Reinert T Henriksen TV Christensen E Sharma S Salari R Sethi H. Analysis of Plasma Cell-Free DNA by Ultradeep Sequencing in Patients With Stages I to III Colorectal Cancer. JAMA Oncol (2019) 5(8):1124–31. doi: 10.1001/jamaoncol.2019.0528 PMC6512280 31070691

[87] Tie J Cohen JD Wang Y Christie M Simons K Lee M. Circulating Tumor DNA Analyses as Markers of Recurrence Risk and Benefit of Adjuvant Therapy for Stage III Colon Cancer. JAMA Oncol (2019) 5(12):1710–7. doi: 10.1001/jamaoncol.2019.3616 PMC6802034 31621801

[88] Tarazona N Gimeno-Valiente F Gambardella V Zuniga S Rentero-Garrido P Huerta M. Targeted Next-Generation Sequencing of Circulating-Tumor DNA for Tracking Minimal Residual Disease in Localized Colon Cancer. Ann Oncol (2019) 30(11):1804–12. doi: 10.1093/annonc/mdz390 31562764

[89] Tie J Wang Y Tomasetti C Li L Springer S Kinde I. Circulating Tumor DNA Analysis Detects Minimal Residual Disease and Predicts Recurrence in Patients With Stage II Colon Cancer. Sci Transl Med (2016) 8(346):346ra92. doi: 10.1126/scitranslmed.aaf6219 PMC5346159 27384348

[90] Parikh AR Van Seventer EE Siravegna G Hartwig AV Jaimovich A He Y. Minimal Residual Disease Detection Using a Plasma-Only Circulating Tumor DNA Assay in Patients With Colorectal Cancer. Clin Cancer Res (2021) 27(20):5586–94. doi: 10.1158/1078-0432.CCR-21-0410 PMC8530842 33926918

[91] Wang DS Yang H Liu XY Chen ZG Wang Y Fong WP. Dynamic Monitoring of Circulating Tumor DNA to Predict Prognosis and Efficacy of Adjuvant Chemotherapy After Resection of Colorectal Liver Metastases. Theranostics (2021) 11(14):7018–28. doi: 10.7150/thno.59644 PMC8171084 34093868

[92] Shoda K Ichikawa D Fujita Y Masuda K Hiramoto H Hamada J. Monitoring the HER2 Copy Number Status in Circulating Tumor DNA by Droplet Digital PCR in Patients With Gastric Cancer. Gastric Cancer (2017) 20(1):126–35. doi: 10.1007/s10120-016-0599-z 26874951

[93] Yang J Gong Y Lam VK Shi Y Guan Y Zhang Y. Deep Sequencing of Circulating Tumor DNA Detects Molecular Residual Disease and Predicts Recurrence in Gastric Cancer. Cell Death Dis (2020) 11(5):346. doi: 10.1038/s41419-020-2531-z 32393783 PMC7214415

[94] Leal A van Grieken NCT Palsgrove DN Phallen J Medina JE Hruban C. White Blood Cell and Cell-Free DNA Analyses for Detection of Residual Disease in Gastric Cancer. Nat Commun (2020) 11(1):525. doi: 10.1038/s41467-020-14310-3 31988276 PMC6985115

[95] Powles T Assaf ZJ Davarpanah N Banchereau R Szabados BE Yuen KC. ctDNA Guiding Adjuvant Immunotherapy in Urothelial Carcinoma. Nature (2021) 595(7867):432–7. doi: 10.1038/s41586-021-03642-9 34135506

[96] Cai Z Chen G Zeng Y Dong X Li Z Huang Y. Comprehensive Liquid Profiling of Circulating Tumor DNA and Protein Biomarkers in Long-Term Follow-Up Patients With Hepatocellular Carcinoma. Clin Cancer Res (2019) 25(17):5284–94. doi: 10.1158/1078-0432.CCR-18-3477 31217202

[97] Razavi P Li BT Brown DN Jung B Hubbell E Shen R. High-Intensity Sequencing Reveals the Sources of Plasma Circulating Cell-Free DNA Variants. Nat Med (2019) 25(12):1928–37. doi: 10.1038/s41591-019-0652-7 PMC7061455 31768066

[98] Chan HT Nagayama S Chin YM Otaki M Hayashi R Kiyotani K. Clinical Significance of Clonal Hematopoiesis in the Interpretation of Blood Liquid Biopsy. Mol Oncol (2020) 14(8):1719–30. doi: 10.1002/1878-0261.12727 PMC7400786 32449983

[99] Cristiano S Leal A Phallen J Fiksel J Adleff V Bruhm DC. Genome-Wide Cell-Free DNA Fragmentation in Patients With Cancer. Nature (2019) 570(7761):385–9. doi: 10.1038/s41586-019-1272-6 PMC6774252 31142840

[100] Jin S Zhu D Shao F Chen S Guo Y Li K. Efficient Detection and Post-Surgical Monitoring of Colon Cancer With a Multi-Marker DNA Methylation Liquid Biopsy. Proc Natl Acad Sci U S A (2021) 118(5):e2017421118. doi: 10.1073/pnas.2017421118 33495330 PMC7865146

[101] Nassiri F Chakravarthy A Feng S Shen SY Nejad R Zuccato JA. Detection and Discrimination of Intracranial Tumors Using Plasma Cell-Free DNA Methylomes. Nat Med (2020) 26(7):1044–7. doi: 10.1038/s41591-020-0932-2 PMC8500275 32572265

[102] Nuzzo PV Berchuck JE Korthauer K Spisak S Nassar AH Abou Alaiwi S. Detection of Renal Cell Carcinoma Using Plasma and Urine Cell-Free DNA Methylomes. Nat Med (2020) 26(7):1041–3. doi: 10.1038/s41591-020-0933-1 PMC8288043 32572266

[103] Taiwo O Wilson GA Morris T Seisenberger S Reik W Pearce D. Methylome Analysis Using MeDIP-Seq With Low DNA Concentrations. Nat Protoc (2012) 7(4):617–36. doi: 10.1038/nprot.2012.012 22402632

[104] Chen K Sun J Zhao H Jiang R Zheng J Li Z. Non-Invasive Lung Cancer Diagnosis and Prognosis Based on Multi-Analyte Liquid Biopsy. Mol Cancer (2021) 20(1):23. doi: 10.1186/s12943-021-01323-9 33514352 PMC7844900

